# The Lived Experience of Chronic Hepatitis B: A Broader View of Its Impacts and Why We Need a Cure

**DOI:** 10.3390/v12050515

**Published:** 2020-05-07

**Authors:** Thomas Tu, Joan M. Block, Su Wang, Chari Cohen, Mark W. Douglas

**Affiliations:** 1 Storr Liver Centre, Westmead Clinical School and Westmead Institute for Medical Research, Faculty of Medicine and Health, The University of Sydney, Westmead NSW 2145, Australia; mark.douglas@sydney.edu.au; 2 Hepatitis B Foundation, Doylestown, PA 18902, USA; joan.block@hepb.org (J.M.B.); chari.cohen@hepb.org (C.C.); 3 Center for Asian Health, Saint Barnabas Medical Center, Florham Park, NJ 07039, USA; Su.Wang@rwjbh.org; 4 World Hepatitis Alliance, London SE1 3YD, UK; 5 Centre for Infectious Diseases and Microbiology, Marie Bashir Institute for Infectious Diseases and Biosecurity, University of Sydney at Westmead Hospital, Westmead NSW 2145, Australia

**Keywords:** lived experience, Hepatitis B virus, stigma, discrimination, HBV cure, chronic hepatitis B, liver disease, psychosocial impact, community, patient experiences, quality of life, public health, socioculture, elimination

## Abstract

Chronic hepatitis B (CHB) is one of the most widespread liver diseases in the world. It is currently incurable and can lead to liver cirrhosis and cancer. The considerable impacts on society caused by CHB through patient mortality, morbidity, and economic loss are well-recognised in the field. This is, however, a narrow view of the harms, given that people living with CHB can be asymptomatic for the majority of their life-long infection. Of less-appreciated importance are the psychosocial harms, which can continue throughout an affected person’s lifetime. Here we review the broad range of these impacts, which include fear and anxiety; financial loss and instability; stigma and discrimination; and rejection by society. Importantly, these directly affect patient diagnosis, management, and treatment. Further, we highlight the roles that the research community can play in taking these factors into account and mitigating them. In particular, the development of a cure for hepatitis B virus infection would alleviate many of the psychosocial impacts of CHB. We conclude that there should be a greater recognition of the full impacts associated with CHB to bring meaningful, effective, and deliverable results to the global community living with hepatitis B.

## 1. Introduction

Chronic infection with the hepatitis B virus (HBV) is the most common blood-borne infection and the major cause of liver disease worldwide. It affects almost 300 million people worldwide and causes 884,000 deaths each year from liver cancer and cirrhosis [1,2]. While ~4% of the world’s population lives with chronic hepatitis B (CHB), its prevalence is not uniformly spread. The World Health Organisation-defined regions of Africa, the Western Pacific, Europe, the East Mediterranean, South East Asia, and the Americas have estimated CHB prevalence rates of 8.8, 5.3, 3.0, 2.1, 1.9, and 0.8% respectively [3]. There is marked variation in prevalence between and within countries of each region, with CHB disproportionately affecting people living in poor socioeconomic areas and vulnerable populations (e.g., people who are incarcerated and injecting drug users), likely due to inadequate access to health services and greater risk of exposure [4,5,6,7,8,9].

The majority of chronic HBV infections are caused by newborn exposure to the virus soon after birth (e.g., mother-to-child fluid exchange during the birth process). Chronic HBV infection can also occur by horizontal transmission (e.g., through unprotected sexual contact; sharing of razors, toothbrushes, or injecting equipment; or non-sterile tattooing, dental, or surgical procedures). Generally, the diagnosis of CHB is made years to decades after the initial infection due to a long asymptomatic phase (often lasting until the late stages of liver disease when limited treatment is available). Even if a diagnosis is made early, the risk of disease progression is not completely removed but is only reduced by current treatments (which suppress virus replication without clearing the infected cells) [10]. There is presently no cure for CHB.

In 2016, the World Health Organization (WHO) called for the global elimination of viral hepatitis by 2030. The majority of people with CHB live in countries that now have national viral hepatitis plans [11]. Many of these plans encompass population-specific communications campaigns to improve awareness and promote testing [12]; community-based programs to provide testing and linkage to care to under-served and high-risk communities [13,14,15]; and pilot projects integrating HBV testing and care into health systems to improve the capacity of primary care providers to test and manage people with CHB [15,16,17,18,19].

As only ~10% of all those infected are aware of their HBV status [2], many interventions are focused on increased testing. Other key components of HBV elimination include increasing rates of birth dose vaccination and catch-up vaccination for adults, improving the infrastructure of treatment and access to care, simplifying guidelines, and curing HBV infection.

The development of a cure is seen as a top priority in the HBV research field [20,21,22]. Cure research has been justified and driven mostly by those working in research, health care, and public health. However, little formalised input has come from the viewpoints of the primary stakeholders: the individuals living with CHB themselves. Currently, the affected community has only limited opportunities to give direct feedback to those researching and developing new therapies. For example, recent key reviews and perspectives describe the need for a cure for HBV [20,21,22], but only mention patient experiences in terms of tolerance or preference to specific curative therapies.

This neglect of lived experience ignores the true impact of CHB, which is greater than the simple sum of mortality and morbidity figures. We believe that researchers can benefit from understanding the lived experience of people with CHB, even if considered in purely utilitarian terms (Appendix A—Why should the patient perspective be heard?):

- To clarify the rationale for finding a cure (e.g., “Why does a cure matter?”);
- To understand if proposed treatment interventions would be useful (e.g., “Is the cure that I am proposing going to be practical in the real world?”);
- To avoid any unintentional harm to the affected community (e.g., by exacerbating stigma);
- To maintain a trusting and respectful relationship between the scientific community and affected communities.

Therefore, we aim to provide a glimpse into the tapestry of issues that affect those living with CHB, beyond the direct physical disease. As authors, we speak with unique perspectives that span the spectrum of HBV scientific and clinical expertise and experiences. Three of us live with CHB and are experts in the field as well: TT is a molecular biologist studying different forms of HBV DNA in the infected liver and how they contribute to the persistence of infection; SW is a primary care physician specializing in the care and management of HBV; and JB is a nurse who co-founded the Hepatitis B Foundation, a research and disease advocacy non-profit organization. Author MD is an infectious diseases specialist and virologist who regularly treats patients with CHB. Author CC is a public health scientist, developing international programs to increase HBV testing and linkage to care in high-risk communities.

In this manuscript, we review the literature on patient experiences associated with CHB. Throughout the paper, we provide first-hand accounts, which contextualise what these impacts mean in a real-world setting. Our ultimate goal is to inform researchers about the context in which their discoveries would be deployed and, in turn, shape their research questions into those that will most benefit the affected community and society as a whole.

## 2. The Lived Experience of Chronic Hepatitis B

In this section, we highlight the psychosocial impacts experienced by an individual living with CHB due to the actual, potential and perceived harms associated with the disease. We do not focus on impacts on those surrounding the affected person (e.g., friends and family members) or the broader effects on a community scale (economy, legal system, human rights, etc.), though we touch upon these in passing when discussing the direct harms. While we acknowledge that psychosocial issues can affect disease awareness (e.g., understanding that HBV is a serious disease and that testing is important) and engagement with a diagnosis of HBV (transitioning from basic knowledge to health-seeking behaviours), these have been well covered by others [23,24,25,26,27] and are outside the scope of this report.

**Figure 1:**
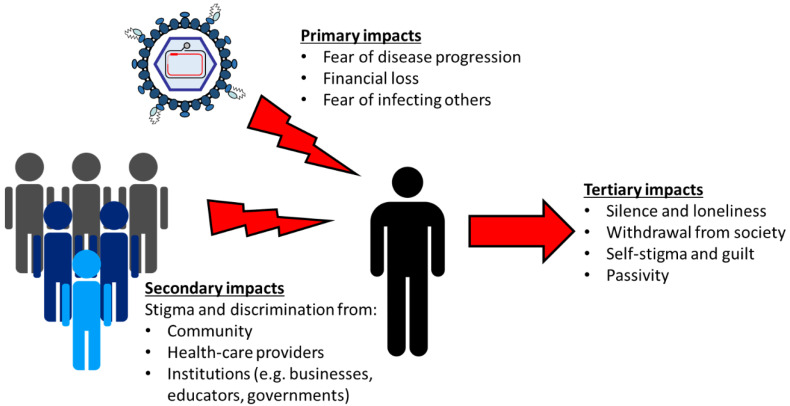
An overview of the psychosocial impacts of HBV infection on an individual living with CHB.

People living with CHB experience the disease as broadly affecting their lives through personal and social impacts [28]. Every person’s lived experience of CHB is unique, but common issues are repeatedly reported and we describe these below. While we accept that patient reality is complex and many of these issues are inter-connected, we have separated our discussion into three sections for the sake of clarity (Figure 1):

1. Primary impacts: the direct psychosocial impacts on the person living with CHB caused by the disease;
2. Secondary impacts: the impact on the affected person caused by the response from society (e.g., family members, healthcare providers, and community and government institutions);
3. Tertiary impacts: the harms associated with an affected person’s response to societal stigma and discrimination.

### 2.1. Primary Impacts

#### 2.1.1. Fear of Liver Cancer and Cirrhosis

**TT:** *Almost 300 million people worldwide have chronic hepatitis B infections and 884,000 people die from its complications every year.*

*Most hepatitis B researchers have heard these figures hundreds of times.*

*Every day I hear these statistics and, even with the emotional distance of being a scientist, it grinds away at me. It’s a daily reminder of what my fate could be.*

*Every day, I find myself wondering—what is that niggling ache in my side? Each morning, I check for the yellowing of my eyes.*

The initial reaction upon a diagnosis of HBV infection is generally one of stress and anxiety about one’s own health and future [29,30,31,32,33,34], which continues throughout a chronic infection as an intermittent worry about disease progression and premature death at a young age. Increased health-related fears have been linked to greater knowledge about HBV, particularly of HBV-related complications (e.g., the possibility of liver cancer) [35,36,37,38]. In contrast, other studies have linked greater anxiety with insufficient information provided by the physician [39]. Thus, striking the right balance of conveying information is important and often depends on a patient’s health literacy, individual personality, and outlook (this has been studied in the context of hepatitis C virus diagnosis [40]).

Published studies [28,35] and our qualitative experiences working with patients [Hepatitis B Foundation, unpublished data] reveal that the quality of information given to recently diagnosed patients and the amount of counselling delivered after patient diagnosis is generally too limited. Patients feel the need for more data on modes of transmission, the natural progression of the disease, complications, and treatment options. As a result of this lack of advice, they report feeling alone, distressed and unprepared to cope with the health implications of their infection. Patient support in the form of post-test counselling (whether from specialized counselling services or clinicians themselves) both at time of diagnosis and throughout the lived experience could lessen this reaction.

Fears about personal health can also be exacerbated by the physical symptoms of the disease. In large, multi-national studies of patient-reported CHB outcomes [41,42], poorer health-related quality of life is consistently related to advanced liver disease. Anxiety and other depressive symptoms are common, particularly as physical symptoms manifest due to advancing liver disease [43,44,45,46]. In contrast, those without symptoms of liver disease report lower (but still present [47]) impacts on mental well-being in relation to health-related matters [41,42].

#### 2.1.2. Financial Loss and Instability

**JB:** *The burden of cost placed on the healthcare system to test, monitor and care for those living with CHB and for those who progress to cirrhosis and/or liver cancer is in the order of billions of dollars per year. But what about the hidden financial costs to patients that are not considered by health economists?*

*For those living with CHB like myself, we must go every 6 months to a liver specialist for a physical exam, blood tests and an ultrasound to monitor disease progression. Even with good insurance, expenses add up and are not necessarily reimbursed: taking off up to a full day of work depending on the location of the clinic, which can result in lost income; childcare costs to keep an appointment; travel and parking costs that can be expensive in a city; increased co-pays for a specialist visit and imaging studies; and even larger co-pays for a brand name drug to control our disease. These are just a few of the out-of-pocket costs that people with CHB can expect to pay during their lifetime.*

**SW:** *Even as a physician who is always counselling HBV patients on their need for care to prevent liver cancer, I don’t often dwell on the fact that I too face this risk. One of the hardest times for me was when my husband and I were getting life insurance. We were surprised at how expensive coverage for me was because I was living with CHB. It made me wonder how the actuaries number-crunched all the scientific studies and medical research to put a price on the chance of my passing.*

Given that HBV is often asymptomatic and the healthcare costs can be exorbitant, many of those diagnosed avoid ongoing clinical monitoring or treatment [32]. For those who do follow through and pay the medical costs, the consequences of a diagnosis of HBV can have a devastating impact on their financial situation.

Patients with CHB can struggle with the out-of-pocket expenditures related to blood tests and imaging needed to assess disease stage [29]. For example, a Chinese study of 894 inpatients with CHB across three cities in Shandong province showed the highly-disruptive financial costs to patients, even to those with insurance (~95% of this study population) [48]. The average annual admission for CHB, decompensated cirrhosis, or HBV-associated liver cancer costs ~40%, 85% or 150% of the average annual household income of the participants, respectively. Notably, this was the out-of-pocket expenditure after insurance reimbursement. In a Beijing cohort, similar estimations for the yearly cost of recommended monitoring for CHB patients were about 10% of the average annual income of Beijing residents [49].

Anxiety about these expensive medical costs are common among those affected. For example, in a large nationwide Japanese study, the majority (~60%) of CHB patients started on antiviral therapy worried about the cost of medications [39]. This worry can drive treatment decisions based on cost instead of medical need (e.g., using a cheaper antiviral with a low barrier to resistance such as lamivudine, rather than more effective drugs such as entecavir or tenofovir [28]). Inadequate treatment can lead to poorer patient outcomes [50,51] and greater expense to the health care system as a whole [52].

The ongoing management and the physical symptoms of CHB can also incur a loss of income, exacerbating financial struggles. The time and inconvenience required for clinical visits can disrupt work, and is often a high barrier for continued follow-up [53,54]. Additionally, traditional breadwinners of a household (i.e., males in their 40s–60s) comprise the main group of CHB patients with symptoms, which can adversely impact a family’s financial stability. Finally, the health care of the affected individual is not the only cost associated with a diagnosis of HBV: a diagnosis of HBV generally prompts an entire family to be tested, vaccinated or treated, resulting in a greater economic burden.

#### 2.1.3. Fear of Infecting Others

**CC:** *I remember taking a phone call from a sobbing young woman who had just given birth to a healthy baby girl. She had not nursed or touched her baby after the delivery and when returning home, she had not even picked her up because she was so afraid of infecting her baby.*

*After four days at home and a frantic search on the internet, she called us to ask whether her doctor’s advice in the hospital was correct: she could potentially infect her baby until her baby received the second dose of the HBV vaccine at two months. I reassured the young mother that the first dose of the HBV vaccine given in the delivery room would sufficiently protect the newborn, and that she should definitely nurse and care for her baby without any fear of possible transmission, as per the recommendations of the U.S. Centers for Disease Control and Prevention.*

Patients with CHB often report being worried about passing the virus on to close contacts and relatives [29,30,34,38,55]. For example, in a 483-person survey in Malaysia, more than half of the individuals after a diagnosis with CHB were worried about spreading HBV infection to family and friends [38]. Correspondingly large proportions were also observed in Australia- and Iran-based studies, with 53% and 80% of participants with CHB respectively listing “spreading HBV to family members” as one of their main concerns [34,36]. These concerns can cause people with CHB to feel shame and guilt, and consider themselves as poor prospects for marriage or starting a family (described more fully below under “Tertiary Impacts”). Studies have demonstrated that people self-stigmatize, distance themselves from family members, choose not to get married, and/or decide not to have children due to this fear [28,30,33,56,57].

The fears about transmission can be exacerbated by poor or incomplete knowledge [30]. In the Malaysian study cited above, about half of the study participants (50.6%) did not share dining utensils and the majority (93.2%) falsely believed that HBV could be transmitted by the sharing of eating and drinking utensils [38]. Being wary about hugging, kissing, and sharing food (despite these not being common routes of transmission) is also a concern in people with CHB as reported in multiple studies [30].

### 2.2. Secondary Impacts

**TT:** *Early on in my career, I went to a party and met a girl. She asked me what I was working on as a research scientist and I answered, hepatitis B. I told her it was prevalent in my community, my family. She asked me directly – do you have hepatitis B?*

*At this point, I had to think and make a judgement about how this person is going to react. My parents came from Vietnam during the war and always have told me to not make waves, keep my head down, and work hard. No politics, no personal incriminating facts to strangers. So what were the consequences of my response going to be?*

*Is she going to respect the honesty?*

*Is she going to refuse to go out on a date with me?*

*Is it going to reinforce the view of “dirty migrants coming to our country taking our health care”?*

*Will authorities somehow be notified and my family thrown out of the country?*

*I chose honesty. I told her the truth. And we’ve just celebrated our four-year wedding anniversary. But this goes to show how much anxiety and fear there is behind every social interaction involving hepatitis B. For others, such honesty may not be so easy.*

The impacts driven by the societal response to someone with CHB chiefly takes the forms of stigma and discrimination [58,59,60]. Stigma (a view held that marks someone as an “other” or of lesser value) and discrimination (the adverse treatment of someone due to stigma) go hand-in-hand. Stigma is often the rationale for discrimination; discrimination can reinforce stigma through decreased opportunities in society. Stigma and discrimination can come from many sources, including the community, healthcare providers, and societal institutions (such as businesses, education facilities, and governments).

#### 2.2.1. Stigma and Discrimination by Community

**MD:** *I have cared for several HBV-positive young women (likely infected at birth) who were devastated when they discovered they had HBV infection, due to incorrect beliefs about likely routes of virus transmission. They could not understand how they had become infected, as they were still virgins and had no history of injecting drug use. Moreover, they were being judged by family and friends as if they had acquired HBV in one of these ways. One young woman had been beaten by her father, ostracised by the whole family and thrown out of her home. In other cases (both men and women), marriages were under threat after receiving a diagnosis of HBV, because of perceived infidelity. It is not widely known that the majority of people with CHB are infected as newborns during the birth process.*

Fear of being infected is the most common driver of HBV-related community stigma [61,62,63,64,65]. Indeed, preventing the risk of infection (e.g., vaccination) has been shown to decrease the level of community discrimination [65]. Stigma is promoted by the lack of accurate knowledge, leading to false perceptions about people with CHB [35,56,61,62,66,67,68,69].

Common false perceptions about HBV include:

- HBV can be spread by sharing food or eating with someone;
- HBV can be transmitted through simple physical contact;
- HBV is only transmitted through promiscuous sex or illicit drug use;
- HBV is caused by dirty conditions;
- HBV is a genetic disease; or
- HBV always leads to death.

As some of these misconceptions are biased toward behaviours that are not broadly acceptable to the community, they present significant barriers to people getting tested or treated. Moreover, these can be harmful in and of themselves, due to the associated discrimination. Multiple studies have found that people with CHB are ostracized by their communities, affecting them in multiple and cumulatively significant ways [28,30,32,33,69,70,71]. Several studies in Beijing and rural China found that people not infected with HBV expressed discomfort in close contact (~45%) or sharing meals (39%–51%) with people living with CHB [70,71]. Respondents did not want their children to play with or marry someone with CHB (~75 and ~90%, respectively [71,72]). They also believed people with CHB should not be allowed to work in restaurants (58%) or in childcare (44%), despite these occupations not being associated with transmission of HBV. The suffering due to this ostracism can be overwhelming and unrelenting given its constant and widespread nature.

Whether increased education about HBV decreases stigma is not clear. Some studies suggest an inverse relationship between stigmatizing behaviors and HBV knowledge, particularly in knowledge about infection prevention and transmission routes [71,73]. However, greater knowledge did not always result in reduced stigma. Providers, patients and family members had a high level of HBV knowledge but still felt they should isolate those with HBV in a study cohort in Haimen City, China [74]. The association between greater knowledge of HBV and increased stigma has been observed in multiple populations [75].

It is likely that the stigma and fear of being infected affect the community at a level beyond an intellectual understanding of the risks. Thus, the messaging (and not only content) of education is important. For example, some have suggested the correction of misunderstandings about inaccurate HBV transmission routes plays a more important role in reducing discrimination than understanding of accurate HBV transmission routes [76,77]. To reduce HBV-related discrimination, policymakers should consider broad and multiple measures with a unified message to decrease the burden of stigma and discrimination, including increasing public education about HBV; promoting universal adult HBV immunization programs; and increasing screening and linkage-to-care programs in at-risk communities.

#### 2.2.2. Stigma and Discrimination by Healthcare Providers

**JB:** *For more than 15 years, I listened to the painful stories of people facing HBV-related discrimination. Many stories involved healthcare students and workers that I related to as a professional nurse. In one memorable case, I counseled a young medical student who was denied permission to continue his clinical rotations after being diagnosed with CHB. This was in 2011 at an accredited U.S. medical school! Although his viral load was reduced with antiviral therapy, the attending physicians would not permit him to work in the hospital in any capacity, so he was forced to quit.*

*In another case, a young female surgeon lost her career because she was diagnosed with CHB. While it took almost 6 months for a panel of hospital physicians and administrators to make the final decision, during this time she was ostracized by her colleagues and felt like “a leper” in the hospital where she had trained.*

Patients are dependent on healthcare providers as trusted sources of health information and of ongoing care. Disappointingly however, healthcare workers can also display stigma and discriminate against people with CHB despite their higher education in medical issues. For example, almost half of surveyed CHB patients in a Vietnamese study reported feeling discrimination by healthcare workers [69]. Moreover, in a multi-centre study in China, patients with CHB perceived or experienced discrimination in their interactions with medical providers and health staff [78]. Respondents were advised not to have children (4.1% of married respondents), to terminate their pregnancy by medical or family planning department staff (4.8% of female respondents), or refused services for family planning or reproductive health (3.3% and 3.2%, respectively).

Lack of accurate and up-to-date knowledge also extends to healthcare workers and can impact patient health outcomes. A questionnaire was sent to 250 registrants of the China National Conference on the Prevention and Control of Viral Hepatitis. The majority of respondents were physicians or health workers, of whom 34% were not aware that CHB is often asymptomatic and 29% did not know that CHB confers a high risk of disease progression to cirrhosis and liver cancer [79].

The reporting of patient test results (as sometimes required for matriculation or pre-employment) can inadvertently promote discrimination: 38% and 25% of physicians surveyed above reported positive HBV results back to patients’ employers and schools, respectively [79]. Because a personal diagnosis is made known to employers and school officials, there is a risk of institutional discrimination for affected individuals.

Providers’ own personal attitudes and lack of knowledge about CHB can also have direct health impacts and lead to inadequate clinical management. First, providers can be disinclined to provide optimal care due to fear of transmission from the patient. For example, 18% of nurses in a national Japanese study agreed or somewhat agreed that they would be reluctant to give care to HBV patients [80]. This attitude was inversely proportional to respondents’ self-reported confidence in their ability to prevent transmission.

Some providers continue to use obsolete terms, such as “hepatitis B carriers” to describe people with CHB. This particular term is problematic because it can stigmatize those affected and implies that disease progression risk is low or that no further follow-up or treatment is needed [81]. Such terminology can label a patient rather than focus on the disease state and can result in lack of action towards actively managing and treating people with CHB.

Finally, providers may be unaware that effective treatments are available or that HBV treatment guidelines have changed, so may not appreciate the importance of regular monitoring and treatment for CHB. Indeed, providers’ knowledge in screening [82,83] and management [84,85] of CHB is suboptimal even in high-resource countries, such as Australia and the US. Without active management and appropriate treatment, patients can be at even greater risk for progressing to cirrhosis and/or liver cancer and dying prematurely [86,87]. The harm for patients as a result could be two-fold: the disease itself and the anger, guilt, and depression from the realisation that life-threatening liver damage could have been prevented.

#### 2.2.3. Stigma and Discrimination by Institutions

**JB:** *I was diagnosed with CHB after a routine employee physical when I changed nursing jobs. One day at work, I received a call from the employee health doctor that completely upended my life. I went into her office and she got straight to the point: “You have hepatitis B.” My mind went blank and I felt numb. She continued speaking but I didn’t hear a word. The Scream by Edvard Munch reflected exactly how I felt. After stumbling out of the office into the nearest bathroom and throwing up, I was convinced that I was going to die.*

*To better understand the context of my severe reaction, it was in the late 1980s when HBV and HIV patients were both kept in isolation rooms and I had to gown, glove and wear eye goggles to provide care. Two of my recent patients had died painful deaths from liver cancer, one of whom was a nurse in her late 30s. This is why I thought HBV meant death.*

*Once my nurse manager was informed of my HBV diagnosis, I was immediately suspended from my job and had no idea if I would be able to return. To make matters worse, my child was unceremoniously kicked out of the hospital-based day-care and could not return unless his paediatrician wrote a note saying he could provide a “100% guarantee” that the staff would be safe from possible infection. I was absolutely devastated. Not only did I feel betrayed by my hospital, I felt that I had betrayed my husband and young child.*

**CC:** *At the Hepatitis B Foundation, we have heard many stories of people living outside the U.S. who have lost spouses, children, jobs and educational opportunities after a diagnosis of HBV. In the U.S., healthcare students and workers have faced particularly egregious institutional discrimination. In 2011, we successfully advocated for and participated in updating the U.S. Centers for Disease Control and Prevention’s 20-year old guidelines for HBV-infected healthcare students and workers. These updated guidelines then became the cornerstone of a lawsuit we filed against a state medical school that had denied admission to two affected students. With the leadership and help of the U.S. Department of Justice, we won the case in 2013. As a result of the court’s decision, HBV infection became a protected condition under the Americans With Disability Act, a federal law that prohibits institutional discrimination based on a person’s disability, such as CHB.*

There is widespread institutional discrimination encountered by people with HBV that affects multiple aspects of their lives, including their opportunities for education, employment, and residency/citizenship. In a Chinese study, three quarters of people with CHB reported experiencing discrimination because of their hepatitis B infection [78], affecting their education and employment choices and economic opportunities. Indeed, China had enacted laws in the 1980s (which have since been repealed) that limited access to education and employment for people living with CHB [28].

Many countries simply do not provide legal protection against discrimination, and people with CHB can lose their jobs, be forced to leave school, or be denied childcare. Even if legal protections exist, poor enforcement of these laws mean stigma and discrimination continue [28,88]. Despite recent legislation in China that makes it illegal to discriminate based on HBV status, a study based in China showed that 40% of CHB patients reported undergoing coerced (unlawful) pre-employment HBV testing and 29% of these individuals thought that they lost job opportunities because of their HBV status [70]. As a result, some people with CHB pay bribes to avoid testing, work under poorer conditions in smaller companies that do not test, pay others to take their blood test for them, or struggle to find work at all [28].

Medical institutions (where knowledge of transmission risks and prevention should be higher than among the general public) can also display such discrimination. In our direct experiences in the United States and Australia, students with CHB have been denied admission to medical, dental and nursing schools or denied clinical placement as part of their training. Medical, dental and nursing professionals have been terminated once their HBV diagnosis is known. These examples of institutional discrimination have been justified by outdated guidelines (requiring proof of immunity against HBV, i.e., serum positive for anti-HBs antibodies) and lack of awareness about the effective treatments for controlling CHB (which significantly reduces the risk of potential transmission to others).

Finally, many countries have immigration limitations, such that people with HBV cannot get visas or can be deported if they are diagnosed with HBV. For example, Australia’s migration laws (which were changed in 2019) rejected permanent residency applications from people diagnosed with CHB on the basis of their cost to the socialised healthcare system. In the United Arab Emirates, guest workers who are diagnosed with HBV are denied work/residence permits [89]. This kind of government-sanctioned discrimination can be devastating and is another poorly-documented harm for people with CHB.

### 2.3. Tertiary Impacts

The person living with CHB can react to community stigma and discrimination in a range of ways. We describe below reactions that have been reported by multiple studies and discuss the impacts they have on the affected individual. We note that this section is broken up for clarity and is not separated into categories that are exclusive (a person may experience many of these mindsets), permanent (one may change their attitudes over time), sequential, or exhaustive (other reactions are possible).

#### 2.3.1. Silence and Loneliness

**SW:** *I’ve had patients who self-stigmatize and fear the repercussions of disclosing their infection, so they keep it to themselves and don’t want to tell their partners or family members. When they do bottle it up, it eats away at them: they live in constant fear of discovery and they bear the burden of the disease alone. I often counsel that they should make the concern for the health of their loved one override their fear of self-protection. Indeed, the ability for their loved one to protect themselves by screening, vaccination, or management might be one of the best gifts they could give them. I encourage people to disclose and, if they struggle with it, I sometimes have them bring their loved ones so we can discuss the diagnosis together and answer any questions. Because I recognize their loneliness, I will often share that I also have the infection, so they feel less alone and know there are many of us, and that we can live fruitful lives.*

*On my own journey, it has been life-changing for me to meet other people living with hepatitis B and share our journeys together. I have met many fellow patients from working with the World Hepatitis Alliance and the Hepatitis B Foundation, and if they were not in my life, I would not have felt emboldened to tell my own story. This kind of connection and camaraderie is really important and something we need to build within the affected community. We must be visible and be engaged in decision making, research and policies that affect us. I feel that as a community we must be the voice for so many who are alone and feel they don’t have a voice. As we do so, we create a support system for ourselves to combat stigma and discrimination.*

The experienced (or expected) stigma and discrimination can be handled in multiple ways. One strategy to mitigate these harms (or their potential harms) is to simply hide one’s HBV status, limiting exposure to stigma and discrimination [35]. Indeed, experienced stigma has been inversely associated with patients disclosing their HBV status, with some reporting regret after revealing this information to others [70].

To avoid the responsibility of informing a partner of their HBV status (and facing potential rejection), it is not uncommon for people with CHB to simply eschew the pursuit of intimate relationships [28]. The fear of never being able to marry or start a family can be devastating. Indeed, in Chinese studies, those with CHB reported feeling that they were undesirable as spouses (33% vs. 17%) and that they brought trouble to their families (58% vs. 34%) more often than uninfected people [70,71]. Maintaining silence about one’s HBV status is likely to exacerbate the other psychosocial impacts described above (e.g., by restricting available emotional and social resources to an affected individual). However, by its nature, it is difficult to measure the true extent to which this silence affects the mental health of the affected community.

#### 2.3.2. Withdrawal from the Majority (or Mainstream) Society

**TT:** *In Australia, CHB has been framed as a migrant disease (despite HBV infection being present in Australia even before its colonisation by the British). I feel a responsibility to not reinforce this stereotype.*

*Although I was born in Australia, I am different to what I see in Australian culture (TV, movies), professionals in my life (teachers, doctors) and who’s in power (e.g., representatives in parliament). Whether I like it or not—and whether it is accurate or not—I represent a community that has in the past been marginalised, and, in some cases, still is. My parents were part of the first major wave of Asian migration after the White Australia Policy was repealed in 1973. My HBV infection is part of a tapestry of issues that mark me and single me out, and it affects how I interact with people in life. Being open with my HBV diagnosis has the potential to differentiate me from a “True Australian” and reinforce the trope of the “diseased migrant, who is sponging off the government.”*

*While I personally have not been treated poorly compared to others in less-supported situations, it is easy to see how one might feel the need to withdraw from a society that doesn’t view you as an equal person, but instead as a burden to be reluctantly tolerated.*

As the majority of CHB cases in low-prevalence countries occur in marginalised communities (e.g., indigenous communities, migrants, incarcerated people, etc.), the false association between poor hygiene and HBV has even greater impact. The stigma of HBV can then be extended into justifying the rejection of the poorly-represented minority groups from the majority society. Thus, CHB can act as a justification for existing discrimination (based on race, class, socio-economic status, etc.) and distance minority groups from the majority society. This can amplify the existing harms associated with poor inter-community relations, such as disenfranchisement, a sense of not belonging, and discrimination.

This distancing can also lead to poorer health outcomes. Some marginalised populations already tend to see the clinical services of the majority society as last resorts. For stigmatizing conditions such as CHB, many may prefer to avoid clinical services and see traditional or alternative health practitioners [54,76], who are not likely to be trained in the management of CHB and so are unable to prevent the HBV-associated disease progression.

#### 2.3.3. Guilt and Self-Stigma

**JB:** *Being diagnosed with chronic hepatitis B was devastating personally. Not knowing whether I had infected my husband and child or not was absolutely traumatizing. It took more than a week to find out their test results; for those 8 days, I lived with a terrible guilt that I had unknowingly spread the virus to those whom I loved the most.*

*Since then, I have spoken with hundreds of patients who have shared similar stories, though they did not always have happy endings. What I’ve learned is that so many of us experience needless guilt for having contracted HBV (since most of us were infected at birth), and yet, we live with a self-imposed label and anxiety of being “infectious.” Compounding the guilt and shame of being chronically infected with HBV, many people don’t want to share their diagnosis with others for fear of losing relationships with their loved ones and friends, being fired from their jobs, or even splitting up their marriages.*

*After 30 years, I decided to break my silence and publicly share my CHB story. Living in a closet, like millions of other people like me, had become an enormous obstacle to fully enjoying life. Telling the truth was such a liberating experience. I felt free from guilt, and, yes, even shame. I felt unafraid. I finally felt whole.*

People living with CHB too often accept the idea that they deserve the stigma and discrimination they receive from the community. This mindset can be justified by reasons that are secular (e.g., “I was involved in high-risk activities”) or religious (e.g., karmic retribution, a punishment for “sin”, etc.). Assuming personal blame for infection can precipitate harmful feelings of guilt and self-stigma.

With this attitude, the affected individuals have conceded to the underlying assumption that they are worth less because of their HBV status, which can be exacerbated by the language used in describing them (e.g., as a health or economic “burden”). This low self-worth can lead to poorer health outcomes. For example, people with low self-worth may not feel that they deserve to be treated [32], particularly if it comes at the expense of the financial stability of their household.

#### 2.3.4. Passivity

Another strategy to cope is to simply ignore the disease altogether. Many cultures and religions are linked with the idea of fate, so getting tested or treated is not necessary—“whatever will be, will be.” Moreover, the absence of active treatment can also play a role in patient passivity. If there is no treatment plan offered at the time of diagnosis, patients often skip their clinical follow-up appointments and miss the opportunity to start effective antiviral therapy if they fall within treatment guidelines [35]. Even with antiviral therapy, the risk of liver cancer remains [10], which adds to the feeling that nothing can be done and the urgency of starting treatment can be muted. Some may even forgo diagnostic testing, arguing that if nothing can be done to treat the disease, then they would rather not know. The development of a cure for HBV would certainly help overcome this passive attitude (Appendix B - Actions that researchers can take).

## 3. The Impact and Development of a Cure for CHB

Despite the availability of an effective vaccine and approved therapeutic treatments, stigma and discrimination against people with CHB continues in our society. These attitudes are chiefly related to the perceived risk of infection and the incurable nature of CHB, and so they will likely persist until a cure for CHB is developed. In order to effectively eliminate the primary and secondary (and by extension, the tertiary) impacts as we have described above, an ideal cure should halt liver disease progression, prevent transmission of the virus after cessation of active treatment, and be accessible by all people living with CHB. If such a clinical outcome could be achieved, much of the psychosocial harms and suffering associated with CHB could be mitigated; many might realise the hope of being accepted fully by their communities.

There is a consensus among expert clinicians and scientists that this clinical outcome could be achieved by the clearance of circulating HBV surface antigen, a so-called “functional cure” [90,91]. There are multiple therapeutic approaches currently being pursued to induce a “functional cure” of CHB (extensively reviewed in [92,93,94]), including inhibiting production of virus products; inducing the antiviral immune response; stopping new liver cells from being infected; and/or actively destroying the virus DNA in an infected cell. A “functional cure” is not the complete removal of all traces of HBV (e.g., covalently closed circular DNA and integrated DNA forms of the virus may still be present in a functionally cured patient). However, it remains the most realistically achievable outcome given current investigational therapies in the pipeline.

Whether the state of “functional cure” inhibits disease progression and viral transmission to an acceptable level is still under considerable debate in the field. It is our view that people living with CHB should have representation in this conversation, so that their values, expectations, and desires are heard. Greater acceptance rates, uptake rates, and effectiveness could be achieved if there is broad agreement with all stakeholders.

## 4. Limitations of Our Review

People living in Asia and those of Asian descent living in high-resource countries have been well represented in the published literature on CHB, as reported by previous systematic reviews [58,59]. While the same psychosocial issues we have described above are also observed in less frequently reported studies of African and Middle-Eastern communities [27,32,33,59,77], there are likely cultural differences that affect the perceptions and stigma associated with CHB. Since few studies have directly compared different communities, exactly how different cultures experience CHB remains insufficiently characterised.

In general, the perspectives from the majority of people living with CHB worldwide are still poorly represented, particularly from those who do not access the health care system. We have also limited our review to English language articles, while most people with HBV do not live in majority English-speaking countries. In some languages, there may not be even be a word for the disease [95], severely hampering the ability to study community perceptions and lived experiences.

## 5. Conclusions

In summary, the impact of CHB is much broader and greater than the physical disease itself. Indeed, a larger proportion of people with CHB are affected by the associated psychosocial burden than by the physical symptoms. The research and personal anecdotes presented above highlight the fact that CHB occurs not in a vacuum, but affects an individual living within a society. The development of a therapeutic cure for HBV would facilitate the elimination of these less-recognised harms, as well as the HBV-associated liver disease.

Distinct from the scientific limitations in developing such a therapy (as outlined in Section 3), this review highlights multiple issues that present barriers to the elimination of HBV, including stigma and discrimination, resulting in a retreat from society; inadequate disease knowledge and health care management; and the financial costs related to diagnosis, monitoring, and treatment. To change this situation and ensure the maximum impact of future discoveries, we believe that researchers have the responsibility (and the power) to bring together the scientific and the affected communities into a productive relationship (Appendix B – Actions that researchers can take).

A fundamental requirement for engagement is listening to all voices to better understand why a cure is needed and the full extent of how it would help our communities. We are on the road to a cure for CHB, but the exact path is not known. People with CHB should be enthusiastically invited on this journey; studies and therapies should be designed with the needs of the affected community in mind, and with respectful consultation when these needs are not known. By considering all of the impacts that CHB has on the lived experience, we can, as a connected community, develop more meaningful, effective, and deliverable care to the 300 million people living with hepatitis B around the world.

## Appendix A. Why Should the Patient Perspective Be Heard?

### Appendix A.1. To Fully Answer the Question “Why Does a Cure Matter?”

Prevalence, morbidity and mortality figures and economic impact are widely quoted as rationales for the necessity of a cure for CHB. This narrow view is imposed by the scientific and medical community. Understanding the lived experiences of people with CHB is critical to comprehending the full extent of the disease’s impact. For many people living with CHB, the psychosocial impacts (e.g., fear, stigma, discrimination, withdrawal from relationships, rejection by society, and passivity) can occur independently of the physical symptoms and disease progression. Importantly, the psychological and social suffering would be relieved by a therapeutic cure for CHB and should be acknowledged when justifying the need for drug discovery and development.

### Appendix A.2. To Inform Researchers What a Useful Cure Looks Like

Patient-centred issues affect the uptake and implementation of any new diagnostic and therapeutic measure. If global elimination of HBV is to occur, then the consideration of patient issues that directly affect uptake rates of testing and treatment is as important as any new technological innovations. Inviting patient perspectives to research discussions is crucial to understanding potential barriers early on when adjustments can be made more easily. In the worst-case scenario, valuable researcher time, resources, funding, and community goodwill will be wasted if a diagnostic or therapeutic is not be taken up by people with CHB.

### Appendix A.3. To Limit Harm

It is common in the HBV research literature to refer to individuals with CHB in objectifying and dehumanising terms (e.g., targets, hosts, carriers, or as simple figures of global mortality statistics). Phrases such as “non-compliant loss to follow up” have been used as a sterile descriptor of a range of behaviours that are driven by complex societal pressures (such as those discussed in the main text).

In other instances, scientists will emphasise the impact and community health burden of CHB to promote awareness and justify funding. However, depending on how this information is presented, the description of the problem can also spread and exacerbate existing stigma associated with CHB. For example, community stigma will be reinforced if patients are simply described as economic or health burdens; as highly contagious; or as carriers, vectors, and sources of ongoing infection in the community. There is a difference between being a problem and being someone with a problem.

Thus, not being informed about the lived experience of CHB can unintentionally increase the level of stigma and discrimination. This has a direct impact on community health; affected patients may be discouraged, disinclined, or disempowered from pursuing diagnosis and treatment. By taking greater care in understanding patient experiences, researchers can avoid inadvertently working against their own interests. In their privileged roles as experts in the community, researchers should realise that their words have power and that there is an obligation to wield them responsibly.

### Appendix A.4. To Maintain a Trusting Relationship with the General Public

Given that basic discovery research (and the salaries of its practitioners) are largely dependent on public funding and trust, it is of utmost importance for science and scientists to maintain a strong relationship with the broader community.

At the same time, scientists are becoming more visible in the wider community. With rising levels of research communication and open access publication, both science and scientists are becoming less separated from the public (including the affected community). Thus, there is greater potential to either strengthen or unintentionally upend this relationship. Understanding the affected community promotes the former (increasing empathy and trust), while minimising the latter (deeper insight into what might cause harm or offence).

## Appendix B. Actions that Researchers Can Take

### Appendix B.1. Acknowledge Lived Experiences

Researchers can acknowledge the lived experiences of those with CHB and internalise the concept that the psychosocial harm associated with HBV infection is more than just than the physical symptoms. Citing appropriate studies in publications would serve to draw attention to these psychosocial impacts throughout the field.

### Appendix B.2. Use Appropriate Terminology

The words used by researchers matter. We highlight in the text that the use of inappropriate terms to refer to people living with CHB (e.g., vectors, hosts, carriers, burdens, etc.) can exacerbate the harms associated with HBV infection. In particular, these terms can affect the community’s view of how important the disease is, how people with CHB should be treated, and how patients see themselves. The use of more empowering terminology should be used where possible to limit the amplification of social stigma.

### Appendix B.3. Partner with Affected Communities

Public advocacy is important to increase awareness and help people understand what to do if they are diagnosed with CHB. There are generally fewer people living with CHB serving as public advocates or ambassadors due to widespread stigma (compared to higher profile diseases such as HIV). As scientists are among the most trusted professionals in almost all societies [96,97], they have the power to speak up for those who cannot to help change this culture, and therefore have some responsibility to bring this change about. Scientists can seek to partner with existing civil societies and patient advocacy groups to facilitate greater awareness, increase funding, and promote legislation to improve access to health care and quality of life for the affected community.

This partnership is not necessarily a one-way street. As mentioned in the main text, people living with CHB can provide a greater perspective on the needs of the community at large. Inviting representatives from the affected community into the research process could inspire novel avenues for enquiry and understanding.

### Appendix B.4. Educate Society

As specialists, HBV researchers are in an ideal position to educate the public and healthcare workers about fundamental aspects of the virus. A particular area that could limit discrimination is the accurate knowledge about the primary routes of transmission. For most cases, perinatal or vertical transmission (from infected mother to newborn during delivery) is the primary route of infection. This could help to reduce stigma and increase awareness for testing among people from endemic countries.

There are multiple existing opportunities and formats to do this, including registering as a scientific expert in the media (e.g., publications like The Conversation), or public forums and lectures. Sustained effort in the public arena is needed to change how HBV is perceived. As well as benefiting people living with CHB, such a change in perception can also be beneficial for scientists with respect to the potential for increased funding opportunities.

### Appendix B.5. Develop a Cure

The psychosocial harms described in this review exist despite the availability of treatment and vaccination. These interventions can sometimes reduce patient worry and stigma [98,99], but this is not always the case [100]. The development of an affordable, limited course therapy that induces a clearance of serum hepatitis B surface antigen (HBsAg-seroconversion, or “functional cure”) could make a considerable difference in attenuating the psychosocial impacts of CHB.
